# Promoting Glucose Transporter-4 Vesicle Trafficking along Cytoskeletal Tracks: PAK-Ing Them Out

**DOI:** 10.3389/fendo.2017.00329

**Published:** 2017-11-20

**Authors:** Ragadeepthi Tunduguru, Debbie C. Thurmond

**Affiliations:** ^1^Department of Molecular and Cellular Endocrinology, Diabetes and Metabolism Research Institute of City of Hope, Duarte, CA, United States

**Keywords:** insulin signaling, p21-activated kinase, skeletal muscle, glucose transporter-4, glucose uptake, insulin resistance, pre-diabetes, type 2 diabetes

## Abstract

Glucose is the principal cellular energy source in humans and maintenance of glucose homeostasis is critical for survival. Glucose uptake into peripheral skeletal muscle and adipose tissues requires the trafficking of vesicles containing glucose transporter-4 (GLUT4) from the intracellular storage compartments to the cell surface. Trafficking of GLUT4 storage vesicles is initiated *via* the canonical insulin signaling cascade in skeletal muscle and fat cells, as well as *via* exercise-induced contraction in muscle cells. Recent studies have elucidated steps in the signaling cascades that involve remodeling of the cytoskeleton, a process that underpins the mechanical movement of GLUT4 vesicles. This review is focused upon an alternate phosphoinositide-3 kinase-dependent pathway involving Ras-related C3 botulinum toxin substrate 1 signaling through the p21-activated kinase p21-activated kinase 1 and showcases related signaling events that co-regulate both the depolymerization and re-polymerization of filamentous actin. These new insights provide an enriched understanding into the process of glucose transport and yield potential new targets for interventions aimed to improve insulin sensitivity and remediate insulin resistance, pre-diabetes, and the progression to type 2 diabetes.

## Introduction—The Pivotal Role of Glucose Transporter-4 (GLUT4) in Glucose Homeostasis

Predictions indicate that by the year 2050, one in three persons will be diagnosed with diabetes (1). This is based upon trending increases in diabetes and pre-diabetes incidences. At present, estimates suggest that 30–40% of United States adults can be classified as pre-type 2 diabetic (1). Pre-diabetes develops most often as the result of peripheral insulin resistance, a condition of disrupted glucose homeostasis. Proper maintenance of glucose homeostasis requires the coordinated actions of insulin-secreting pancreatic beta cells and insulin-responsive peripheral tissues. Peripheral tissues include skeletal muscle and adipose, whereby insulin stimulates muscle and fat cells to take up the excess circulating glucose. Insulin also triggers the liver to abate its output of glucose. Skeletal muscle and fat cells internalize circulating glucose *via* the insulin-stimulated translocation of GLUT4 storage vesicles (GSVs) from intracellular storage pools to the plasma membrane (PM); upon deposition of the GLUT4 protein on the PM, glucose uptake is increased by 10- to 20-fold (2–4). Studies of tissue-specific/conditional GLUT4 knockout (KO) mouse models have shed light upon the relative contribution in each tissue in isolation to overall glucose homeostasis. For example, skeletal muscle-specific GLUT4 KO mice exhibited severe insulin resistance and glucose intolerance at an early age (5). Similarly, adipose-selective depletion of GLUT4 in mice led to impaired glucose tolerance and hyperinsulinemia (6). As determined from human clamp studies, the skeletal muscle clears 80% of postprandial serum glucose (7–9). In both mouse and human, failure of the skeletal muscle to appropriately respond to insulin is a hallmark of pre-diabetes, insulin resistance, and T2D.

Exercise increases glucose uptake by ~50-fold to meet the increased energy demands of muscle during physical activity [reviewed in Ref. (10)]. Glucose uptake into skeletal muscle is mediated through three processes: glucose delivery, glucose transport, and the metabolism of glucose after its entry into the cell. The exercise-induced increase in blood flow to skeletal muscle increases glucose and insulin delivery to the skeletal muscle, along with recruitment of capillaries which increases the surface area for glucose delivery (11–13). The levels of GLUT4 expression influence glucose transport, differing in the diverse skeletal muscle fiber types [e.g., higher GLUT4 expression levels in the type I oxidative fibers (soleus muscle)] compared with the glycolytic type II fibers (epitrochlearis and extensor digitorum longus) (14–16). Longer duration exercise is implicated in the conversion of type II to type I muscle fibers (17), increasing the efficiency of the muscle in metabolizing glucose; i.e., type I muscle fibers produce more ATP (being oxidative goes through mitochondria) than glycolytic (anaerobic glycolysis) which produces less ATP per glucose molecule. Importantly, exercise-induced glucose uptake was preserved in insulin-resistant skeletal muscle in which insulin failed to promote glucose uptake, emphasizing exercise as a key therapeutic intervention for metabolic diseases. Indeed, many efforts focus on the “exercise in a pill” approach to combat the looming pandemic of pre- and T2D. However, simulating the beneficial effects of exercise, or restoring insulin responsiveness, with the intent to reinstate robust GLUT4 trafficking and glucose uptake requires intimate knowledge of the signaling and trafficking itinerary of GLUT4 vesicles in skeletal and fat tissues. This review will focus upon the advances in our understanding of the insulin signaling pathways that lead to GLUT4 vesicle trafficking/mobilization in skeletal muscle.

## GLUT4 Mobilization to Sarcolemma and T-Tubules of Skeletal Muscle

**Figure 1:**
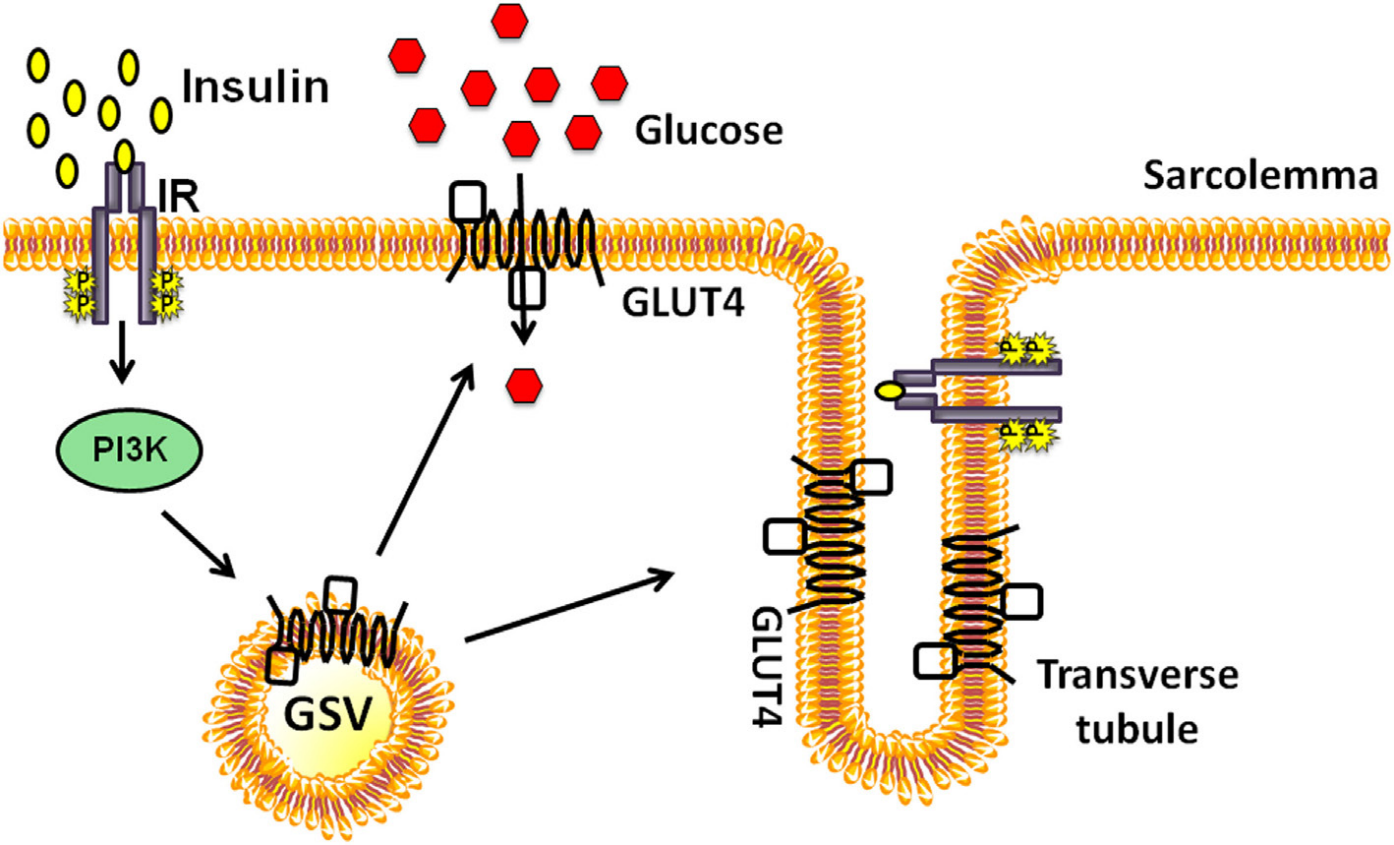
Insulin-stimulated glucose transporter-4 (GLUT4) vesicle translocation to the skeletal muscle transverse tubules and sarcolemmal membranes. Insulin binds to the extracellular subunits of the insulin receptor (IR) that activates the phosphoinositide-3-kinase (PI3K) signaling cascade and stimulates the translocation of a pool of GLUT4 storage vesicles (GSVs) to both transverse tubule and the sarcolemmal membranes.

The number of GLUT4 proteins integrated into the PM depends on the rate of endocytosis and exocytosis of GSVs. Early studies demonstrated that acute insulin injection into the hindlimb muscle of rats resulted in a surge of GSVs to the PM fractions, accompanied by a decrease in the intracellular GSVs (18). PM fractions in muscle are comprised of sarcolemma and T-tubule membranes, both of which are recipient target membranes of insulin-induced GSVs; GSV’s are otherwise principally housed in perinuclear regions under resting conditions (Figure 1). Interestingly, exercise similarly increases GLUT4 content in the PM fractions (19). Moreover, exercise-sensitive GLUT4 transporters were found not to originate from the insulin-sensitive intracellular membrane fraction; these data pointed to the existence of two distinct pools of intracellular GLUT4 transporters (19). Further bolstering the two-pool concept were reports showing the additive effects of exercise and insulin treatment on glucose uptake by rat skeletal muscle (20, 21). The underlying assumption has been that signaling to the exercise- or insulin-sensitive GSV pools to evoke their particular translocation to the PM was distinctly different. Indeed the Rab GTPase activating protein AS160 (also known as TBC1D4) is considered a key part of the insulin-stimulated signaling mechanism; its paralog TBC1D1 is not essential for insulin-stimulated signaling, but its phosphorylation is substantially increased by exercise [reviewed in Ref. (22)]. This review will focus largely upon new events in insulin-stimulated signaling pathways leading to GSV translocation to the PM.

## The Trafficking Itinerary of GLUT4: From Exocytosis to Endocytosis, and Back

### Exocytosis

**Figure 2:**
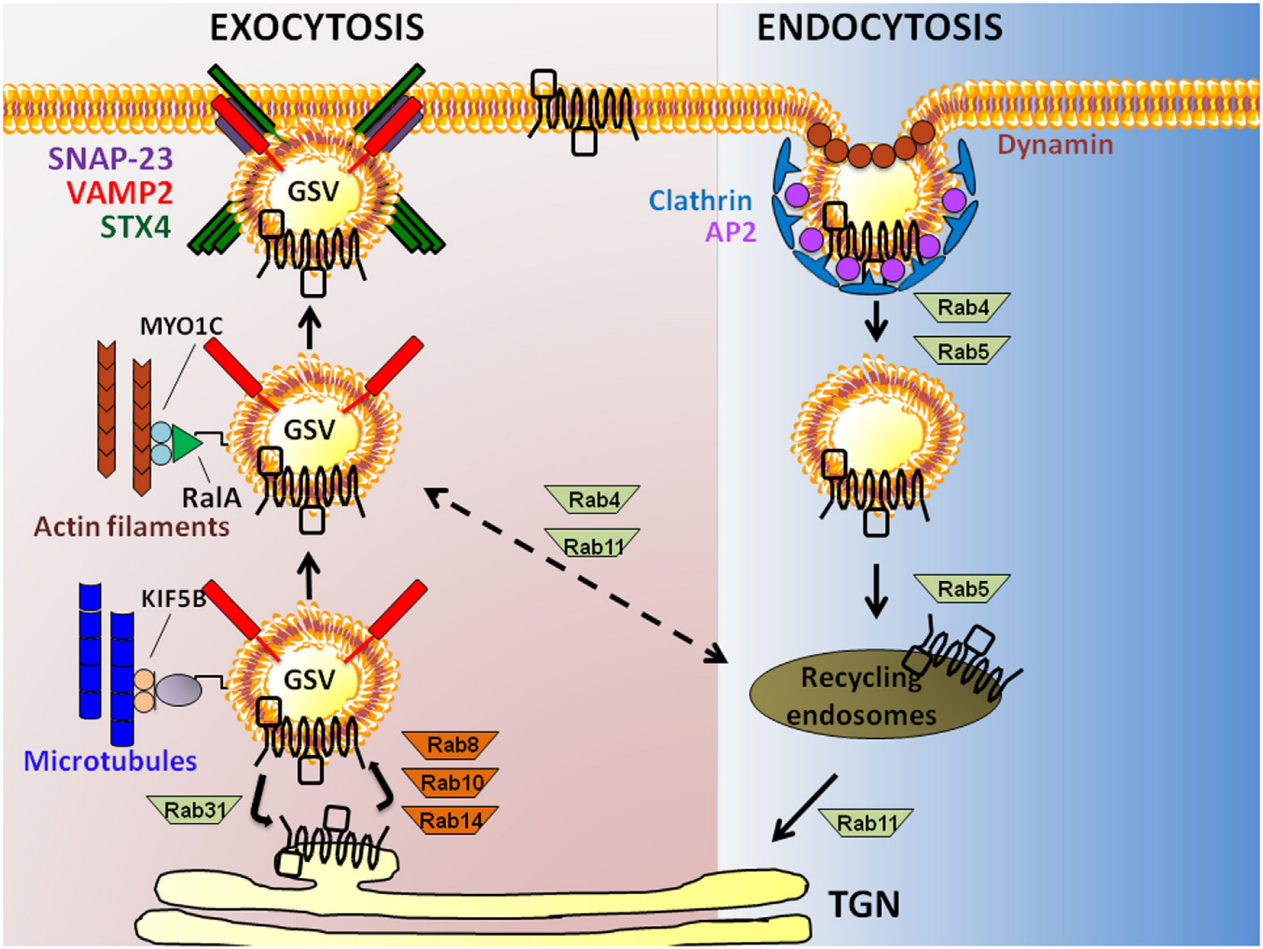
Exocytosis to endocytosis, and back. Schematic model showing actin filament and microtubule-dependent exocytosis of glucose transporter-4 (GLUT4) (leftmost pathway), and clathrin-mediated GLUT4 endocytosis (rightmost pathway) in skeletal muscle and adipose cells. GLUT4 present at the plasma membrane (PM) is endocytosed in a Clathrin-dependent manner requiring the adaptor protein-2 (AP2) adapter and the GTPase Dynamin. Rab GTPases 4 and 5 are implicated in the distal step to generate recycling endosomes carrying GLUT4, afterwhich GLUT4 is taken to the trans-Golgi network (TGN) *via* Rab11 or possibly back to a GLUT4 storage vesicle (GSV) pool *via* Rab4/11. Exocytosis of GLUT4 in GSVs that also carry the vesicle SNARE [vesicle associated membrane protein-2 (VAMP2)] from the TGN requires Rabs8/10/14; Rab31 counteracts this action. GSVs travel along microtubules *via* kinesins (KIF5B) and actin filaments (*via* RalA and MYO1C), bringing GSVs into close proximity with the PM t-SNARE proteins [Syntaxin 4 (STX4) and synaptosomal-associated protein-23 (SNAP-23)].

The exocytosis of GSVs requires their translocation to the PM and tethering/docking at PM sites, prior to fusion with the PM. Insulin causes a 10- to 40-fold increase in glucose uptake by facilitating GSV exocytosis (23). Translocation of GSVs is proposed to initiate in response to an insulin signal that triggers release of GSVs from TUG (tether containing a ubiquitin regulatory X domain of GLUT4). Endoproteolytic cleavage of TUG liberates intracellularly sequestered GSVs from a retention pool, permitting their translocation (24). In skeletal muscle, this involves the activations of small GTPases Rab8 and Rab14 (Rab10 is used by adipocytes) (25) (Figure 2). GSVs can also be directed to alternate intracellular regions, where they cycle between GSV pools and the trans-Golgi network (TGN); this aspect is activated by Rab31 (26). GLUT4, along with insulin-regulated amino peptidase, sortilin, and low-density lipoprotein receptor-related protein-1, forms a multimeric protein complex that is packaged into releasable GSVs (27). Insulin triggers the inactivation of Rab31 coordinate with the activation of the small GTPase, ARF6 to increase GSV release from the endosomal recycling compartment (26).

Glucose transporter-4 storage vesicles, like most other secretory vesicles, use the actin-based myosin for translocation across short distances [i.e., less than 1 µm to the PM (28)]. GSVs use microtubule-based kinesins to travel longer distances from perinuclear regions to the PM. Myosin and kinesin motor proteins implicated in these short and long distance travel processes include MYO5A and MYO1C (29, 30), and KIF5B (31), respectively. In adipocytes, the myosin-motor protein MYO1C recognizes the small GTPase RalA present on the GSV membrane, and transports the GSV on the actin cytoskeletal tracks to the docking/targeting site on the PM [reviewed in Ref. (32)]. The GTP-loaded RalA transports GSVs to the targeting site *via* its interaction with exocyst subunits (SEC5 and EXO84). The exocyst subunits are part of a larger exocyst complex, composed of eight distinct proteins (Sec3, Sec5, Sec6, Sec8, Sec10, Sec15, Sec4, and Exo70). This exocyst complex is stabilized at the PM by the insulin-induced activations of protein kinase B (PKB)/AKT and the small GTPase TC10, along with the action of the lipid raft-localized synapse-associated protein 97. Subsequent to this docking step, the protein kinase C-dependent phosphorylation of SEC5 inhibits the interaction of RalA and the exocyst complex, disengaging the GSV from the targeting/docking machinery to prepare the GSV for fusion with the PM. MYO1C is also required for GSV trafficking and glucose uptake in skeletal muscle cells (30, 33), although whether the role of RalA and the exocyst complex is conserved remains unresolved.

Glucose transporter-4 storage vesicle fusion requires SNARE complexes. SNARE complexes are comprised of two target-membrane (t-SNARE) proteins and one vesicle-membrane (v-SNARE) protein. GSV fusion entails the assembly of the v-SNARE isoform vesicle associated membrane protein-2 (VAMP2) and t-SNARE proteins Syntaxin 4 (STX4) and synaptosomal-associated protein-23 (SNAP-23) [reviewed in Ref. (34)]. This cluster of SNARE isoforms differs from that which is classically associated with neurotransmitter release from the presynaptic membrane of neurons (e.g., STX1, SNAP25, and VAMP2), but does overlap with those used by insulin-secreting islet beta cells (e.g., STX1-4, SNAP-23/25, and VAMP2) [reviewed in Ref. (35)]. Docking and fusion of GSVs also requires the recruitment of SNARE accessory proteins to the PM, such as double C2-like domain-containing protein β (Doc2β) (36–40), Syntaxin 4-interacting protein (Synip) (41, 42) and Munc18c (43–48). Doc2β is a positive activator of STX4 in skeletal muscle cells, coordinating the activation/opening of STX4 to promote SNARE complex assembly (49). Synip and Munc18c are both STX4 binding factors that, when overexpressed, exert negative effects upon STX4 engagement in SNARE complex assembly (41–43, 47, 50). However, endogenous Munc18c appears to be limiting for GLUT4 fusion because KO mice show skeletal muscle insulin resistance and deficient GLUT4 accumulation in sarcolemmal and t-tubule membranes (46). Once STX4 is activated, it participates in the SNARE complex formation leading to GSV exocytosis. In addition, data suggest that both Doc2β (51) and STX4 (52–54) can interact with cytoskeletal factors, which may be required as part of the mechanism underlying GLUT4 vesicle fusion with the PM.

### Endocytosis

The two major endocytosis pathways of GLUT4 are clathrin-mediated endocytosis and cholesterol-dependent endocytosis (55). Historically, clathrin was assumed to be the major operating pathway in skeletal muscle cells; however, a study in L6 myoblasts demonstrated that depletion of cholesterol could exert a notable inhibition of GLUT4 internalization and endocytosis (55), lending support for cholesterol-dependent endocytosis as a required process in skeletal muscle cells. Regarding the mechanisms involved in clathrin-mediated endocytosis, Adaptor Protein-2 (AP2) binds to GLUT4, and recruits clathrin to the PM. Here, AP2 packages GLUT4 into clathrin-coated vesicles that pinch off from the PM with the help of the GTPase Dynamin (56). Newly formed vesicles/endosomes that carry GLUT4 are transported from the PM to the cell interior by the microtubule-based motor protein dynein that attaches to the GSVs *via* Rab5 (57). Endocytosed vesicles fuse with sorting endosomes that are either subjected to degradation or recycling. The GLUT4 transporter can be recycled back to the PM by budding off into GSVs from recycling sorting endosomes with the help of Rab4, Rab5, Rab11, and RalA (32).

## The Classic Insulin Signaling Pathway in Skeletal Muscle

Initiation of the classic insulin signaling pathway in skeletal muscle cells begins with insulin binding to the insulin receptor (IR) present on the extracellular surface of the skeletal muscle cell. Insulin binds to the extracellular α subunit of the tetrameric IR, which elicits in the intracellular β subunit both a conformation change and receptor autophosphorylation, ultimately increasing the catalytic kinase activity of the IR, resulting in the recruitment of its receptor substrates [reviewed in Ref. (58, 59)]. Recruited IR substrates include insulin receptor substrate (IRS) *via* pleckstrin homology (PH) domains and phosphotyrosine binding domains in the IRS amino termini. In skeletal muscle, IRS-1, but not IRS-2, plays a critical role in myoblast differentiation and glucose metabolism (60, 61). Downstream of IRS-1, the pathway branches into at least two major transduction routes (62). In one pathway, tyrosine phosphorylation of IR induces IRS-1 to bind Src-homology-2 (SH2) domain-containing proteins including p85, the regulatory subunit of class I phosphatidylinositol-3 kinase (PI3K), targeting PI3K to the PM. At the PM, PI3K phosphorylates phosphatidylinositol 4,5-biphosphate (PIP2) to produce the lipid second messenger phosphatidyl 3,4,5-triphosphate (PIP3) that recruits several PIP3-binding proteins to the PM. In the second pathway, phosphorylated IRS-1 interacts with the adaptor protein complex composed of growth factor receptor bound protein-2 (Grb2) and son-of-sevenless, activating the Ras-MAPK cascade and mitogenesis. The IRS-1-PI3K pathway, which mediates insulin’s action on glucose metabolism, further bifurcates into at least two parallel signaling pathways downstream of PI3K [AKT vs. Ras-related C3 botulinum toxin substrate 1 (Rac1)-p21-activated kinase 1 (PAK1)].

**Figure 3:**
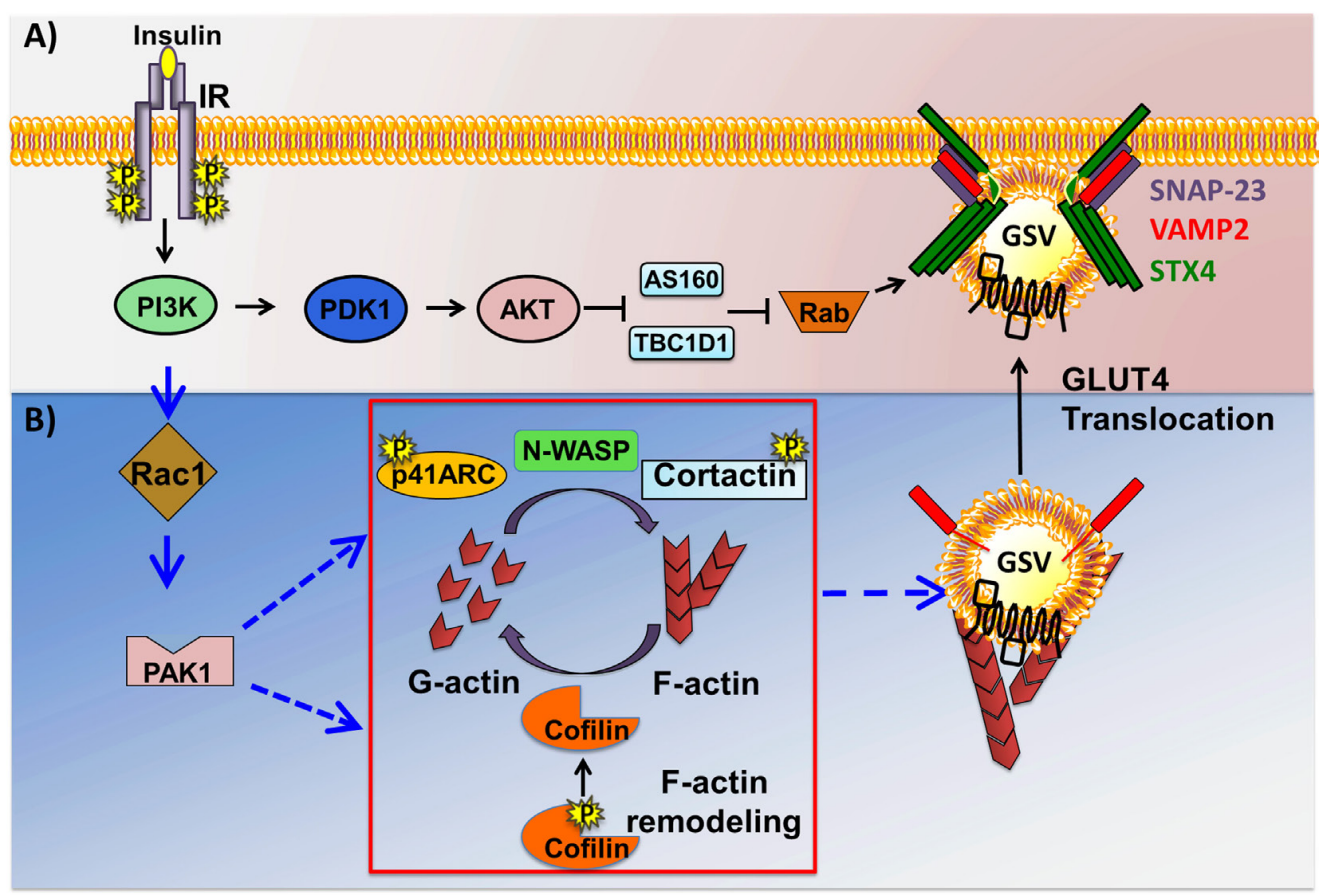
Two arms of the insulin signaling pathway in skeletal muscle cells. Insulin binding to the insulin receptor (IR) results in the activation of phosphoinositide-3-kinase (PI3K), downstream of which the pathway bifurcates into at least two signaling pathways just downstream of PI3K: the canonical AKT→Rab-dependent pathway, and the non-canonical Ras-related C3 botulinum toxin substrate 1 (Rac1)→p21-activated kinase 1 (PAK1)-dependent actin remodeling pathway. **(A)** Activation of phosphoinositide-dependent-kinase 1 (PDK1) triggers it to phosphorylate and activate AKT. Activated AKT in turn phosphorylates and inactivates AS160 and TBCID1, thus facilitating Rab-GTP mediated glucose transporter-4 (GLUT4) storage vesicle (GSV) translocation. **(B)** Activated Rac1 downstream of PI3K recruits its downstream effector PAK1. Activated PAK1 triggers phosphorylation of p41-ARC and its interactions with *N*-WASP and cortactin, promoting filamentous actin (F-actin) polymerization. PAK1 activation also triggers actin depolymerization *via* cofilin, effectively generating the globular actin (G-actin) substrate required for F-actin polymerization, a cyclic process referred to as actin remodeling. Cortical actin remodeling facilitates GSV translocation to and fusion with the cell surface with the help of SNARE complex proteins, vesicle associated membrane protein-2 (VAMP2), Syntaxin 4 (STX4), and synaptosomal-associated protein-23 (SNAP-23), facilitating glucose uptake by the insulin-responsive skeletal muscle cells.

The insulin-stimulated recruitment of p85 by IRS-1 brings p110, the catalytic subunit of PI3K to the PM, where it catalyzes the phosphorylation of the 3′ position on the inositol ring of the phosphoinositide (PI) lipids (63). PI3K specifically catalyzes formation of PIP2 from PI(4)-phosphate. The PIP3 generated from PIP2 then aides in the recruitment and activation of PIP3-binding proteins phosphoinositide-dependent-kinase 1 (PDK1) and AKT (Figure 3A). Of the three AKT/PKB isoforms (AKT1, 2, and 3), AKT2/PKBβ has been shown to be essential for glucose transport into skeletal muscle, using whole body KO rodent models (64) as well as functional studies in skeletal muscle with *in vivo* electrotransfer of constitutively active-AKT2 (65). PIP3-guided PM recruitment of PDK1 and AKT2 aide in the phosphorylation and activation of AKT2^T308^ and of PDK1 and PDK2 (also known as mammalian target of Rapamycin complex 2) (66, 67). Fully activated AKT2 phosphorylates one of its substrates, the Rab guanine nucleotide activating protein (GAP) called AS160^T642^. AS160 targets Rab8A and Rab14 in skeletal muscle (68) and inactivates each Rab. Through its GTPase activating domain, AS160 maintains Rab proteins in an inactive GDP-bound state under basal/unstimulated conditions (69). Upon dissociation from AS160, Rab proteins are switched to their active GTP-bound state to facilitate GSV trafficking to the PM. Interestingly, AS160 is also regulated by the action of AMPK, which is a key regulator of the contraction-induced glucose uptake pathway (70), placing AS160 at a point of convergence linking insulin- and contraction-mediated signaling pathways that facilitate glucose entry into skeletal muscle. Interestingly, these studies also uncovered the importance of the Rho family GTPases and their requirement in signaling to the cytoskeleton to evoke GLUT4 vesicle trafficking. This mechanism is discussed in the next section.

## The New RAC1-p21-Activated Kinase 1 (PAK1)-Actin Remodeling Pathway

In the last decade, much emphasis has been placed on delineating an alternative signaling pathway involving small Rho family GTPases, a pathway that proceeds in parallel to the AKT-AS160 pathway (Figure 3B). Rac1 is a small Rho family GTPase that acts as a molecular switch to execute diverse cellular processes including cell cycle regulation, cell–cell adhesion, and actin cytoskeleton regulated motility [reviewed in Ref. (71)]. Rac1 switches between an active GTP-bound form and an inactive GDP-bound form, *via* actions of guanine nucleotide exchange factors (GEFs), e.g., P-Rex1 (72), -activating factors (GAPs), and -dissociation inhibitors. In skeletal muscle cells, the activation of Rac1 transmits signals to various downstream effectors involved in cytoskeletal remodeling. Although both AKT and Rac1 act downstream of PI3K to facilitate insulin-dependent glucose uptake in skeletal muscle, signaling pathways involving each protein were suggested to take parallel and independent routes (73). This was supported by studies using dominant negative mutants and pharmacological/chemical inhibitors of AKT (74), none of which showed any effect upon insulin-induced actin cytoskeletal remodeling. Consistently, neither Rac1 knockdown nor expression of the constitutively active Rac1 mutant altered insulin-stimulated AKT phosphorylation [reviewed in Ref. (75)]. A recent paper shows in an inducible skeletal muscle-specific Rac1 KO mouse the requirement for Rac1 in exercise-induced GLUT4 translocation (76). Although most evidence shows independent activation of Rac1 and Akt downstream of PI3K, there are reports of AKT2 regulating Rac1 activity to impact GLUT4 translocation in skeletal muscle cells (77, 78), involving the guanine nucleotide exchange factor FLJ00068 (79, 80). Rac1 superactivation has also been shown to activate AKT to trigger induce GLUT4 translocation in a manner independent of insulin (81). Nevertheless, studies using both cell culture models, as well as whole body KO mouse models, have implicated Rac1 and its downstream signaling to the PAK1 to be crucial regulators of both insulin- and contraction-induced cytoskeletal remodeling as a key element in regulated GLUT4 vesicle trafficking (82, 83). Indeed, PAK1 carries the potential to serve as a hub for interactions, acting as a scaffolding platform to dock signaling effectors, and cytoskeletal factors, as discussed in the following subsections.

### Structure, Activation, and Function of PAK1

**Figure 4:**
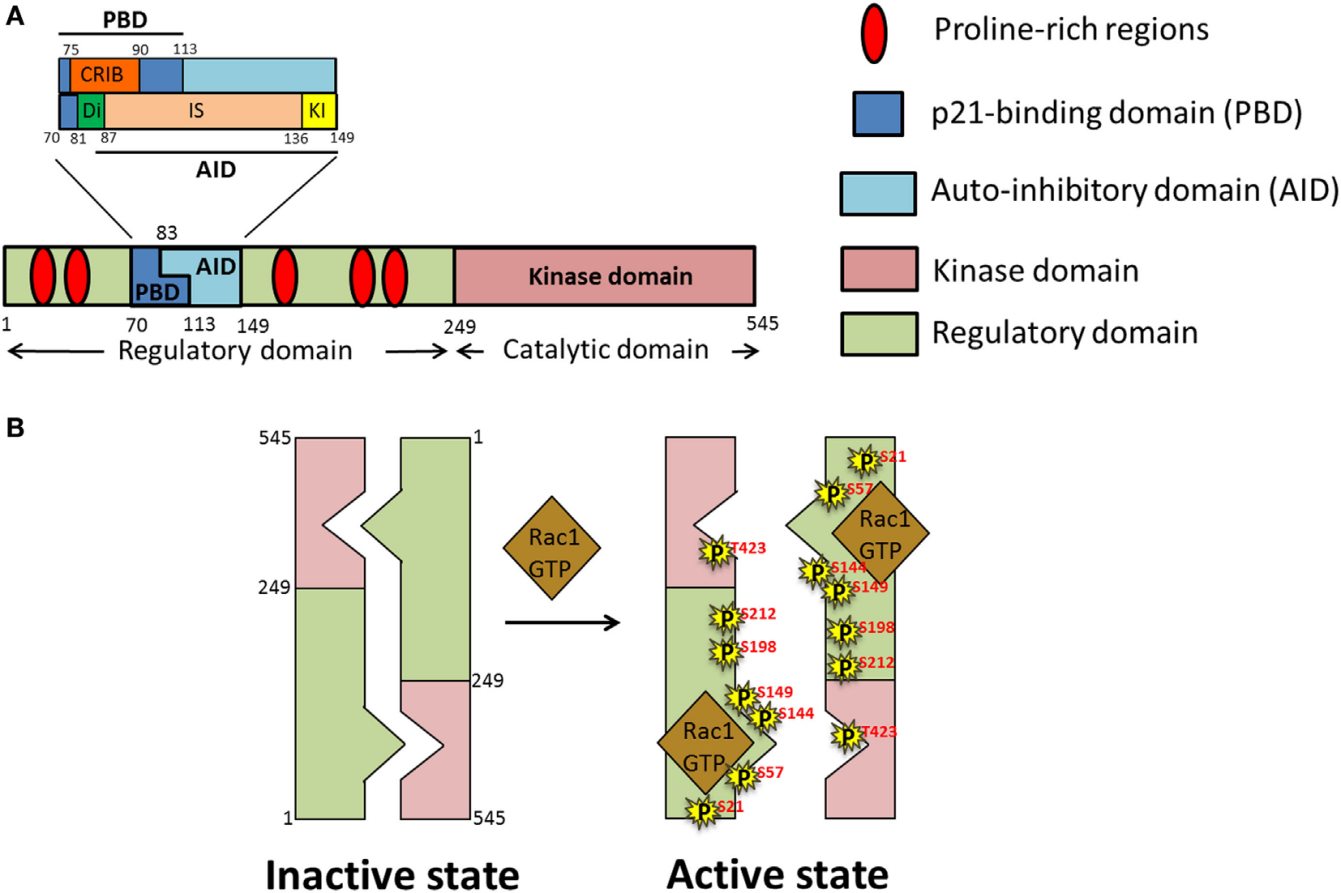
Domain structure and activation of p21-activated kinase 1 (PAK1). **(A)** Schematic representation of different domains of PAK1 polypeptide chain; PAK1 is divided into two main domains, regulatory and kinase. Within the regulatory domain is the p21-binding domain (PBD), comprised of the Cdc42-Rac Interactive Binding (CRIB) and Di domains as well as part of the inhibitor switch (IS) domain. The IS constitutes the bulk of the autoinhibitory domain (AID), along with the addition of the *C*-terminal-most kinase-inhibiting (KI) domain. **(B)** Under basal conditions PAK exists as a homodimer in an autoinhibitory conformation which upon stimulation by growth factors opens up into an active monomeric conformation due to binding of activated small Rho GTPases such as GTP-loaded Ras-related C3 botulinum toxin substrate 1 (Rac1)/Cdc42 and autophosphorylation (P) of PAK on numerous serine (S) and threonine (T) residues.

The PAK family of proteins consists of six serine/threonine kinases that are divided into two groups; homodimeric Group I PAKs (PAK1, 2, and 3), and monomeric Group II PAKs (PAK4, 5, and 6). The basic structure of Group I PAKs consists of an *N*-terminal regulatory domain, which includes a GTPase binding domain [also known as a Cdc42-Rac Interactive Binding (CRIB) domain], overlapping with an autoinhibitory domain (AID), and a *C*-terminal kinase domain (Figure 4A). Group I PAKs form inactive homodimers, where the AID domain of one monomer is bound to the kinase domain of the other. Activation of Group I PAKs occurs upon binding of small GTPases Cdc42 or Rac1 to the CRIB domain, relieving the inhibitory conformation and triggering the autophosphorylation at the activation loop (A-loop) for subsequent activation of PAK’s kinase activity (Figure 4B). Group II PAK activation differs from that of Group I PAKs, since the AID of Group II PAKs is thought to allosterically modify the constitutively phosphorylated kinase so that it becomes active (84). Both Group I and II PAKs are important regulators of various biological processes including cell motility, survival, proliferation, and cytoskeletal organization. Owing to their involvement in the above physiological processes, deregulation of functional PAKs are implicated in multiple inflammatory disorders such as diabetes, cancer, mental retardation, allergy, inflammatory, and cardiovascular diseases (85).

#### PAK1 Proline-Rich Regions

p21-Activated kinase 1 interacts with multiple substrates depending on unique cellular conditions through its five proline-rich regions, which contain PXXP (where P and X represent Proline and any amino acid, respectively) sites that resemble src-homology-3 (SH3) domain binding motifs. Nck, an adaptor protein that contains one SH2 domain and three SH3 domains, interacts with the first proline-rich SH3 domain binding motif (amino acid residues 12–18) of PAK1 through its second SH3 domain. This interaction links PAK1 and receptor tyrosine kinases (such as IR) in cell proliferation and growth (86).

Similarly, PAK1 binds to the Grb2 adaptor protein *via* the second SH3 binding motif of PAK1, mediating the coupling of activated epidermal growth factor receptor, and PAK1 upon EGF stimulation in HaCaT cells (an immortalized human keratinocyte cell line). In this manner, the upstream receptor kinases and downstream PAK1 signaling are linked (87). PAK1 interactions can also occur *via* its non-canonical proline-rich residues. For example, PAK1 binds to the GEF for Cdc42, PAK-interactive exchange factor/cloned out of library protein through a non-canonical proline-rich region (residues 182–203) (88). This ability to bind to GEFs gives PAK1 tremendous versatility in small GTPase signaling.

#### PAK1 Autoregulatory Fragment

The *N*-terminal region of PAK1 contains the regulatory domain (amino acids 1–249) (Figure 4A). This region contains two main segments, a p21-binding domain (PBD) and an AID, collectively called the autoregulatory fragment. The PBD (residues 70–113) includes the minimum sequence for CRIB (residues 75–90), which is essential for binding of the small GTPases. Studies using site-directed mutants and truncated fragments revealed the sequences required for the high-affinity binding of PAK1 to Cdc42 and Rac1 (89–91). The AID (residues 83–149) acts as an inhibitory switch (IS) and is essential for the *trans*-autoinhibition of group I PAKs (92). The *C*-terminal extension of the IS region sequesters the critical element, named the kinase inhibitor (KI; residues 136–149) segment, which passes through the cleft of the kinase domain, and is required for the “autoinhibited state of PAK.” A segment of PAK1 containing the deletion of amino acids 81–87 in the *N*-terminal region of the IS motif, termed as Dimerization (Di; residues 81–87) segment, exhibits a decreased Dimerization (93), implicating an importance of trans-Dimerization for the inhibition of PAK1 under basal conditions. Binding of activated Cdc42 or Rac to the CRIB region relieves the autoinhibited state of the PAK1 dimer by disrupting the intermolecular interactions between the IS domain of one monomer and the kinase domain of the other. Details of the activation process are described in the next section.

#### PAK1 Kinase Domain

The kinase domain (residues 249–545) is the catalytically active site of PAKs and has the standard, two-lobe structure characteristic of all the protein kinases (94). Interestingly, PAK1 contains an intrinsically active kinase domain that catalyzes the serine/threonine phosphorylation of its substrates, even in the absence of activators. This unique feature suggests that tight regulation of the active site is required to avoid unwarranted activation of kinase function. Notably, a stretch of 30 residues in the large lobe of the kinase domain, designated as the “A-loop,” contains the critical autophosphorylation site (T423) required for the complete activation of PAK1 (95). Moreover, evidence for the existence of the kinase domain in an active conformation, even in the absence of T423 phosphorylation, stems from studies using kinase-dead (K229R), and phosphomimetic (T423E) mutants of PAK1 (96).

### Multistage Activation of *Trans*-Autoinhibited PAK1

p21-Activated kinase 1, also known as PAKα, was first discovered in 1994 in rat brain as a protein kinase that binds to the active GTP-bound form of small GTPases Cdc42 and Rac1 (97, 98). PAK1 is the most extensively studied protein among the PAKs, and is highly homologous to the yeast protein Ste20p. The activation of PAK1 is a multistage process that starts with the binding of GTP-bound form of Cdc42 or Rac to the *N*-terminal CRIB of PAK1, which triggers a series of conformational changes involving disruption of the PAK1 dimer and rearrangement of the kinase domain into a catalytically competent state (Figure 4B). Binding of activated Cdc42 or Rac induces conformational changes in the CRIB and unfolds the IS domain, which results in the dissociation of the KI fragment (within the AID) from the catalytically active kinase domain. However, PAK1 assumes its fully activated state only after phosphorylation of T423 and T212 (i.e., autophosphorylation), and subsequent phosphorylation of serine residues S21, S57, S144, S149, and S198 (93) (within the KI region and the IS domain). Phosphorylation prevents the re-Dimerization of PAK1 into an inactive state (92). The significance of the T423 site in the activation of PAK1 comes from the studies showing reduced kinase activity of PAK1 carrying the T423A mutation (99). In skeletal muscle of obese and T2D human subjects, phosphorylation at T423 is reduced (82).

### PAK1 As a Signaling Kinase

Since the discovery of PAK1 in 1994 (98), PAK1 has been found to signal downstream to numerous substrates participating in cytoskeletal remodeling. For example, the ARP2/3 complex, filamin A, cortactin, cofilin, and LIM-kinase (LIMK) are major regulators of the actin cytoskeletal network that can be regulated by phosphorylation by PAK1. In addition to modulating proteins involved in the actin cytoskeletal network, PAK1 also regulates microtubule dynamics. PAK1 phosphorylates tubulin cofactor B, a cofactor in the assembly of α/β-tubulin heterodimers (100). Although studies using agents that inhibit microtubule polymerization (e.g., colchicines) suggested against a requirement for microtubule-mediated regulation of insulin- or contraction-stimulated glucose transport in skeletal muscle (101), more recent findings using mice deficient in carboxyl terminus of HSC70-interacting protein (CHIP) revealed the significance of microtubule dynamics for insulin-dependent GLUT4 translocation in skeletal muscle (102). Decreased GLUT4 translocation was accompanied by reduced microtubule polymerization and phosphorylation of stathmin (at Ser16), a microtubule-regulating protein in the CHIP-depleted skeletal muscle cells. Given that PAK1 phosphorylates stathmin at Ser16 and regulates its activity in Hep2 cells (103), the above findings point toward a potential role of PAK1 in the regulation of microtubule-dependent GLUT4 trafficking in skeletal muscle. Whether decreased phospho-stathmin (S16) levels associate with decreased PAK1 activity or with phospho-PAK1 levels in the CHIP-depleted skeletal muscle cells remains an open question.

### PAK1 As a Scaffolding Protein

Although a number of phospho-proteins have been identified as PAK1 substrates, increasing evidences using kinase-dead mutants of PAK1 indicate a kinase-independent scaffolding function for PAK1 in the regulation of cytoskeletal reorganization and cell motility (87, 95). As a scaffold, PAK1 signals to downstream effectors not by mere transfer of a phosphate group, but by creating a platform to allow the tethering of multiple members of a signaling pathway into complexes.

p21-Activated kinase 1, through its scaffolding function, induces the dephosphorylation of the phosphatase PP2A to increase its phosphatase activity (104). PP2A is a serine protein phosphatase that is involved in glucose transport and glycogen synthesis (105, 106). PP2A dephosphorylates the actin-severing protein cofilin at Ser3, resulting in cofilin activation (107). Intriguingly, whole body PAK1 KO mice lack insulin-regulated cofilin activation in skeletal muscle (83). This PAK1-dependency of cofilin dephosphorylation was corroborated in a subsequent study in L6 myoblasts (108). Hence, one possible explanation for the mechanism of cofilin activation by PAK1 would be that PAK1 scaffolds and activates PP2A, leading to the dephosphorylation, and subsequent activation of cofilin, which in turn regulates the actin remodeling process and facilitates the insulin-dependent GLUT4 translocation in skeletal muscle. Alternatively, PAK1 might activate cofilin *via* its signaling function by regulating slingshot (SSH)1/chronophin (a second potential cofilin phosphatase) phosphatase activity (109). While PAK1 can trigger changes to cofilin *via* LIMK in some cell types (110), several studies in skeletal muscle cells show no role for LIMK in mediating the cofilin (108, 111).

### Regulation of PAK1 Activity

#### Activators of PAK1

In many cell types, GTP-loaded small Rho family GTPases such as Rac1 and Cdc42 can activate PAK1 by binding to the CRIB domain at the *N*-terminal region, relieving the autoinhibited homodimer conformation, to activate the monomer form [reviewed in Ref. (112)]. In addition, the Wnt-1 responsive Cdc42 homolog, an atypical small Rho GTPase regulated by Wnt-1 signaling, also activates PAK1 (113). Notably, however, among these GTPases, including the three Rac isoforms, Rac1 is the only small Rho family GTPase known to regulate both the insulin- and the contraction-stimulated glucose uptake into skeletal muscle (82, 114).

The detailed mechanism regarding how Rac1 activates PAK1 in skeletal muscle cells remains unresolved. Clues from other cell types suggest two potential mechanisms centered on binding to distinct regions of PAK1. In one case, the GTPase partners synergistically with PIP2 at the PM to activate PAK1 *via* their respective binding to PAK1’s CRIB domain and an adjacent region rich in basic residues (115). PIP3, the phosphorylated form of PIP2, recruits PH domain-containing proteins and regulates insulin-stimulated GLUT4 trafficking in peripheral tissues (116). Moreover, the PH domain-containing protein CK2α-interacting partner-1 (CKIP-1) was recently implicated in regulating insulin-stimulated PAK1 activation, and in a PI3K-dependent manner skeletal muscle cells (117). If Rac1 and PIP2 coordinate to activate PAK1, then CKIP-1 or other PH domain-containing proteins may constitute the machinery required for insulin-stimulated and PI3K-dependent PAK1 activation and recruitment to the PM in skeletal muscle cells. In the second case, SH3 domain-containing adaptor proteins such as Nck/Grb2 bind to the proline-rich SH3 binding motifs of PAK1, recruiting PAK1 to the PM, and assisting in the activation of PAK1 by small Rho GTPases (86). Since Nck binds to activated IRS-1 *via* its other SH2 binding motif in response to insulin (118), its ability to also activate PAK1 could coordinate the upstream insulin signaling events involving the cell surface receptors with its this downstream event that would couple signaling to actin remodeling (detailed in the next section) and GLUT4 vesicle translocation.

#### Negative Regulators of PAK1

Because uncontrolled PAK1 activation might result in deleterious metabolic effects, mechanisms to ensure appropriate PAK1 activation are essential. Indeed, the skeletal muscle and kidney-enriched inositol polyphosphate phosphatase was found to negatively regulate PAK1 by blocking its scaffolding function in regulating PDK1-AKT2 mediated insulin-stimulated glucose uptake (119). Given that PAK1 activation levels are reduced in skeletal muscle tissues from T2D patients (82), it is speculated that additional negative regulators exist and may be activated under conditions of metabolic stress.

## Actin Cytoskeleton Remodeling and GLUT4 Trafficking

Signaling to and consequent sorting of GSVs from the intracellular regions of the muscle cell for subsequent exocytosis to the docking/fusion sites at the PM requires spatiotemporal regulation of components of the dynamic cytoskeleton. In adipocytes, multiple studies using microtubule network disrupting agents suggest that both actin- and microtubule-based cytoskeletal regulations are required for the insulin-induced mobilization of GSVs to the PM (31, 120). In skeletal muscle cells, the concept of actin cytoskeleton-based GLUT4 vesicle mobilization is well-established (101). For example, depolymerization of filamentous actin (F-actin) by the actin monomer sequestering agent, Latrunculin B exhibits a time- and concentration-dependent decline in glucose transport into muscle cells, implicating the requirement of an intact F-actin network for insulin-induced GLUT4 translocation and glucose uptake (121). Interestingly, knockdown of Myo1c in L6 muscle cells disrupted actin filaments, whereas Myo1c overexpression immobilized those GSVs close to the PM (as detected by total internal reflection fluorescence) (30). As such, a prevailing concept is that the interaction of vesicular Myo1c with F-actin regulates GLUT4 vesicle tethering to the actin cytoskeleton and subsequent insulin-induced GLUT4 vesicle fusion to the PM (30).

### Insulin-Induced F-Actin Remodeling and GLUT4 Vesicle Translocation

Cytoplasmic actin exists in two forms: monomeric globular actin (G-actin) and F-actin. F-actin remodeling encompasses the process of continuous cycles of polymerization of actin at the barbed end of the existing filament, and depolymerization of F-actin to G-actin at the pointed end. Both F-actin nucleating proteins, such as those of the Arp2/3 complex, and the opposing actions of actin-severing proteins such as cofilin and gelsolin, tightly regulate this F-actin remodeling process (122–124) (Table 1). Importantly, it is the insulin-stimulated remodeling of F-actin, as opposed to simply its polymerization, is essential for the translocation of GSVs to the PM of skeletal muscle cells (125, 126). PAK1 is implicated in both the polymerization and the depolymerization of F-actin in insulin-stimulated GLUT4 vesicle translocation and glucose uptake in L6-GLUT4myc myoblasts and myotubes (108, 127).

**Table 1:** Actin regulatory proteins in insulin-stimulated glucose transporter-4 (GLUT4) translocation.

Name	Function(s)	Binding partners?	Mode of activation	Role in GLUT4 translocation	Reference
Actin depolymerizing factor (cofilin)	Actin-severing protein, membrane protrusion, and cell motility	Chronophin, slingshot (SSH), LIMK	Activated by dephosphorylation of S3 by chronophin and SSH	Required in skeletal muscle cells	(111, 128, 129)
Alpha-actinin-4	Actin filament cross-linking protein, binds actin to the cortical cytoskeleton, and its associated proteins	GLUT4, MICAL-L2, Rab13, p85 subunit of PI3K, AKT1	Relieved of autoinhibition upon phospholipid binding	Required in skeletal muscle cells	(130–132)
Arp2/3 complex	Actin nucleation and branching	PAK1, WASP family proteins, cortactin	Activation by phosphorylation of its regulatory subunit ARPC1/p41-ARC at T21 by PAK1	Required in skeletal muscle cells	(127, 133)
Cortactin	Binds F-actin to prompt nucleation of new filaments	Arp2/3 complex, PAK, ERK, Src, WASP, dynamin	Phosphorylation at Y421, Y466, Y482, S405, and S418	Required in skeletal muscle cells	(134)
Gelsolin	Actin capping and severing protein	Nm23h1, STX4	Calcium binding to gelsolin leads to its activation	ND	(54, 135)
*N*-WASP	Strong nucleation promoting factor that binds to Arp2/3 complex to nucleate branched actin filaments	Cdc42, Src family kinases, WASP interacting SH3 protein, GRB2, cortactin	Relieved of autoinhibition by interaction with binding partner	Required in 3T3-L1 adipocytes and skeletal muscle cells	(127, 136)
WAVE-2	Regulates *de novo* actin polymerization	PIR121/Sra-1, Nap1, Abi1/2, HSCP300	Upon release from Rac1 and NCK, WAVE becomes activated	ND	(137, 138)
Tropomodulin 3	Actin-capping protein, negative regulator of cell migration	AKT2	Phosphorylation at S71	Required in 3T3-L1 adipocytes	(139)

*ND, not determined*.

### Actin Polymerizing Proteins

Several actin nucleating/branching proteins function together to regulate the intricate process of actin polymerization, of which the Arp2/3 complex, a branched actin filament nucleator, is implicated specifically in GLUT4 vesicle translocation in skeletal muscle cells (111). Arp2/3 is a 220 kDa complex consisting of seven subunits (ARP2, ARP3, and ARPC1-5). From a structural perspective, the actin-related proteins (ARP2 and ARP3) resemble monomeric actin because together they mimic an actin dimer, initiating the formation of new actin branches on existing F-actin filaments (140). Knockdown of ARP3 or the ARPC2 subunit of the ARP2/3 complex in skeletal muscle cells abrogated insulin-induced F-actin remodeling and GLUT4 translocation to the PM (111). Intriguingly, PAK1 directly phosphorylates p41-ARC/ARPC1, a regulatory subunit of the ARP2/3 complex, to initiate actin remodeling, in multiple cell types including in skeletal muscle cells (127, 141, 142). Although the ARP2/3 complex can initiate branched actin filament nucleation, it requires the actions of nucleation promoting factors (NPFs), such as neural Wiskott–Aldrich syndrome protein (*N*-WASP), WAVE, and cortactin, to stimulate its activity and carry out actin polymerization (Table 1). *N*-WASP has been implicated in insulin-stimulated GLUT4 translocation (127, 136). The selective inhibitor of *N*-WASP, Wiskostatin (stabilizes *N*-WASP in its autoinhibited state) impairs insulin-stimulated glucose uptake into myotubes, -GLUT4 translocation, -actin remodeling, and insulin-dependent associations with actin, cortactin and the ARP2/3 subunit p41-ARC (127) (Figure 4B).

#### Nucleation Promoting Factors

Nucleation promoting factors fall into two categories: type I NPFs that interact with monomeric actin molecules, and type II NPFs that bind to actin filaments. Overexpression of cortactin, a type II NPF, in L6-GLUT4myc cells increased insulin-induced GLUT4 translocation (134). Furthermore, knockdown of cortactin completely blunted insulin-dependent glucose uptake into L6-GLUT4-myc myotubes (134). While these data clearly show a requirement for cortactin in GLUT4 vesicle mobilization, cortactin is considered to be a relatively weak activator of the Arp2/3 complex and requires strong NPFs such as *N*-WASP, also a type II NPF, to impact Arp2/3-mediated actin polymerization. Supportive of a role for *N*-WASP in skeletal muscle cells, Brozinick et al. showed that *N*-WASP localized to F-actin in an insulin-dependent manner, and that this was abrogated by disruption of F-actin by Latrunculin B in mature skeletal muscle (121). In non-muscle cells, PAK1 has been shown to use cortactin as a substrate, phosphorylating cortactin at residues S405 and S418, increasing its association with *N*-WASP, and facilitating F-actin polymerization (143). While this would serve as a mechanistic link to connect GSV mobilization *via* ARP2/3 and *N*-WASP with cortactin, this remains to be thoroughly tested in skeletal muscle cells. WASP family verprolin-homologous protein (WAVE, a type 1 NPF), an *N*-WASP homolog, is implicated in lamellipodial formation upon stimulation in skeletal muscle cells, however, its role in skeletal muscle glucose uptake is not yet explored (144). To date, there are no reports implicating the role of any known type 1 NPFs in the mobilization of GSV to the skeletal muscle cell surface.

#### Actin Stabilizers

Inhibition of *N*-WASP or PAK1 activities results in blunted activation of STX4 (127), suggesting that function of the SNARE machinery is somehow tethered to actin polymerization. Polymerizing actin is also stabilized by proteins such as fodrin, also known as non-erythroid spectrin. Interestingly, fodrin interacts with the t-SNARE protein STX4 in response to insulin and hence is implicated in the fusion of GSVs with the PM in rat adipocytes (53). Moreover, the calpain-induced cleavage of fodrin at the sarcolemma in muscle is associated with Duchenne muscular dystrophy (145), a disease whose early stage is characterized by insulin resistance (146). Increased cleaved fodrin is also found in pancreatic β cells of T2D subjects (147), further linking fodrin to metabolic control mechanisms in glucose homeostatic tissues/cells.

#### Actin Cross-Linking Proteins

Alpha-actinin-4 is a dimeric actin filament cross-linking protein that binds to GLUT4 and regulates the insulin-dependent GLUT4 vesicle fusion to the PM in association with Rab13 and its effector MICAL-L2 in skeletal muscle cells (130). Filamin A is an actin cross-linking protein that can be phosphorylated by PAK1 at Ser2152 and is involved in PAK1-dependent membrane ruffling in mammalian breast cancer cells (148). Once phosphorylated, filamin A interacts with the CRIB domain of PAK1, inducing its further activation and creates a feed-forward loop of activation of PAK1. In skeletal muscle, cross-sectional analyses show the colocalization of filamin A with phosphoinositidylinositol 3,4,5 trisphosphate (PtdIns[3,4,5]P_3_) 5-phosphatase (SHIP-2) in the membrane ruffles, and these are considered to be the sites of insulin-induced GLUT4 translocation (149). Taken together, these findings suggest a possible mechanism involving PAK1 with filamin A and SHIP-2 in GSV mobilization in skeletal muscle.

### F-Actin-Severing/Depolymerizing and Capping Proteins

Actin depolymerizing factor (ADF/cofilin) and gelsolin are the two major actin-severing proteins implicated in the regulation of the skeletal muscle actin filament network (111). In non-muscle cells, cofilin phosphorylation at Ser3 by LIMK inhibits cofilin’s actin-severing ability (150). In contrast, cofilin’s dephosphorylation at Ser3 by the phosphatase SSH will activate cofilin (151). PAK1 KO mice lacked the insulin-dependent dephosphorylation of phospho-cofilin in their skeletal muscle (83), implicating PAK1 upstream of cortactin phosphorylation. This was subsequently confirmed using the PAK inhibitor IPA3, which abrogated the insulin-stimulated changes to cofilin, coordinate with its inhibition of glucose uptake into myotubes, and actin remodeling (108). LIMK was unaffected by PAK inhibition, supporting the mouse PAK1 KO data (83), adding further support to the concept of PAK1 signaling the depolymerization of F-actin in an LIMK-independent fashion. Interestingly, PAK4 has been shown to activate LIMK and inactivate SSH *via* phosphorylation *in vitro*, suggesting a plausible role in the inactivation of cofilin (152). However, the involvement of PAK4 as a potential regulator of cofilin activity in skeletal muscle *in vivo* is yet to be explored.

Two additional ADFs should be considered as potential players in PAK1-mediated actin depolymerization: gelsolin and tropomodulin3 (Tmod3). Gelsolin is expressed ubiquitously (153, 154), and has been implicated as a regulator of STX4 engagement in vesicle fusion (54). Gelsolin mediates calcium-dependent severing, with calcium increasing its affinity for the ADP-associated actin filament to initiate its severing function (155). Tmod3 is regulated by phosphorylation at Ser71 by AKT2 in 3T3-L1 adipocytes (156), which is required for insulin-initiated actin remodeling. All together, the studies of these actin modifying factors (Table 1) link their actions to F-actin cytoskeletal reorganization and to translocation/exocytosis of GLUT4 vesicles to the cell surface of insulin-sensitive skeletal muscle and adipose cells.

## Discussion and Perspectives

Overall, this review encompasses the journey of GLUT4 from TGN to PM with special focus on the actin regulatory machinery that is required for the transportation of the cargo carrying the GLUT4 transporter in insulin-responsive tissues. Although the role of the actin cytoskeletal network in vesicle trafficking has been well-studied in other cell types, the intricate machinery involved in GLUT4 vesicle translocation in skeletal muscle remains relatively understudied. PAK1, a well-characterized key regulator of actin dynamics in many cell types, functions as a central node that regulates diverse cellular activities such as actin cytoskeletal reorganization, cell motility, cell proliferation, and cell division. This multitude of signals from PAK1 to its downstream targets suggests that substantial deviations in PAK1 activity from normal will be deleterious to the cell. Consistent with this concept, PAK1 signaling impairments in skeletal muscle have been correlated with obesity and T2D (82, 83), indicating the significance of PAK1 in both insulin-dependent glucose uptake pathways in skeletal muscle. To date, the focus has been on PAK1, since PAK2 knockdown in L6 skeletal myoblasts was without effect on insulin-stimulated glucose uptake (108). However, given the recent reports suggesting a role for PAK2 in insulin-induced glucose uptake in neuronal cells (157), studies of PAK2 in primary skeletal muscle may warrant further investigation. Regardless, the identification of novel interacting partners of PAK1 that are specifically involved in the insulin-stimulated glucose uptake process, and not in the survival pathway, will provide us with a tantalizing opportunity to identify potential therapeutic targets for T2D without affecting the normal survival/anti-apoptotic pathways.

## Author Contributions

RT and DT jointly wrote the manuscript and designed the figures. In doing so, both authors agree to be accountable for the content of the work.

## Conflict of Interest Statement

The authors declare that the research was conducted in the absence of any commercial or financial relationships that could be construed as a potential conflict of interest.
